# Trizma as an eco-friendly efficient inhibitor for the acidic corrosion of steel: experimental and computational studies

**DOI:** 10.1038/s41598-022-19060-4

**Published:** 2022-09-12

**Authors:** B. A. Abd-El-Nabey, S. El-Housseiny, M. A. Abd-El-Fatah

**Affiliations:** 1 Faculty of Science, Chemistry Department, Alexandria University, Alexandria, Egypt; 2 Faculty of Sciences, Chemistry Department, Jouf University, Sakakah, Saudi Arabia; 3 Faculty of Science, Chemistry Department, Kuwait University, Kuwait, Kuwait; 4 Faculty of Education, Alexandria University, Alexandria, Egypt

**Keywords:** Electrochemistry, Physical chemistry

## Abstract

The inhibition characteristics of Trizma for corrosion of steel in 1 M HCl was investigated using the weight loss, potentiodynamic polarization, electrochemical impedance spectroscopy techniques and the surface techniques XRD,SEM and EDX. The potentiodynamic results indicated that Trizma act as a mixed type inhibitor for steel in 1 M HCl giving efficiently 93.7% percent inhibition for 1 × 10^–2^ mol/L. The electrochemical impedance spectroscopy results showed an increase in *R*_*ct*_ values and decrease in the value of *C*_*dl*_ with increasing the concentration of Trizma indicating that the presence of Trizma in the solution retards the steel corrosion due to the adsorption of its molecules at the steel/solution interface. The XRD and SEM results indicated that the surface of the steel contains Trizma molecules. The DFT method was investigated to correlate the molecular properties of the studied Trizma with the experimental inhibition efficiency. Langmuir, Flory–Huggins isotherm, and the Kinetic–thermodynamic model were used to fit the corrosion inhibition data of Trizma. The results indicated that the Langmuir isotherm does not fit with the experimental results due mainly to the non-ideal adsorption of its molecules at the steel/solution interface. However, Flory–Huggins isotherms, and the Kinetic–thermodynamic model are applicable and showed that the adsorption process of Trizma on the steel surface is cooperative (Chemical–Physical).

## Introduction

Carbon steel is used on a large scale for industrial applications as an engineering and construction material, including also in transportation, power plants, marine structures, chemical processing, and petroleum production and refining. It has high tensile strength and hardness but is significantly more prone to corrosion.

Many methods are available to reduce the impact of steel corrosion, such as cathodic protection, electrochemical methods, and corrosion inhibitors^1–4^. The use of corrosion inhibitors is a highly efficient and economic method. The most effective corrosion inhibitors used in industry are heteroatomic organic compounds^5^, but most of them are non-degradable and toxic which results in environmental pollution upon discharging. Many research efforts were done for replacing them with environmentally friendly materials. The use of environmentally friendly materials, as green corrosion inhibitors was the best solution. Therefore, it was gaining large preference and interest, due to its relating abundant in nature, the safe use. Novel approaches and current trends in the field of inhibition science and technology for corrosion protection of metals and alloys have been discussed in three recent review articles. Abd-El-Naby et al.^6^ in a review on plant extracts as corrosion and scale inhibitors tabulated 44 inhibitors for corrosion of steel in acid media^6^. Yang^7^ in a review on the role of organic and eco-friendly inhibitors on the corrosion mitigation of steel in acid environments tabulated 41 inhibitors ^7^. Pmaveen et al. (2022) in a review on eco-friendly corrosion inhibitors on mild steel in acidic medium tabulated 11 corrosion inhibitors made from plant extracts^8^.

Trizma, with the formula (HOCH_2_)_3_ CNH_2_ is an organic compound. It is used as a component of buffer solutions extensively in biochemistry and biotechnology^9,10^. Due to the presence of one amino and three hydroxyl groups, it acts as a good chelating agent mostly with + 2 and + 3 oxidation states of the first-row transition metals^10,11^. In addition, the specific structure of Trizma makes it as a good reducing agent for the synthesis of various metal nanoparticles specially Au-NPs. This feature is beneficial in wide employment such as quantum dot, spectroscopic enhancers, sensors, nano-structure fabrications, ultrasensitive detectors, and micro imaging methods^12–14^. Furthermore, it can form different Schiff bases which are used as chromatographic adsorbents for different metal ions^15^.

**Figure 1:**
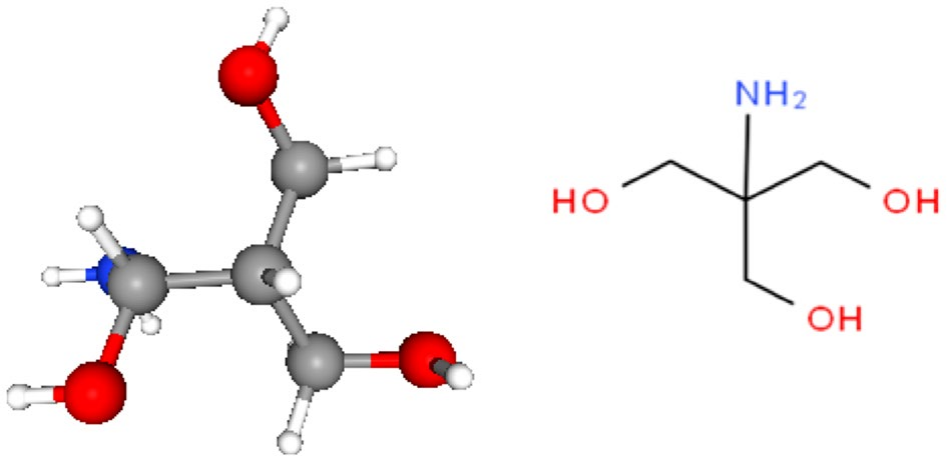
The optimized and schematic structure of neutral Trizma molecule.

In the 1990, researchers turned their interest to using quantum chemical techniques used to cover several important theoretical and computational concepts to provide relevant insight into the molecular/electronic properties of the studied inhibitor, which shows their adsorption onto the metal surface^16–20^. The DFT model has proved to be a common and dependable method for the study of the chemical reactivity of organic corrosion inhibitors^21^. At low cost, it gives vital, exact, and basic parameter values for even hugely complex molecules^22^. It depends on the principle that the energy of a molecule can be determined by its electron density^23^. It was found that the frontier molecular orbitals (MO), including the highest occupied molecular orbital, HOMO, and the lowest unoccupied molecular orbital, LUMO are closely linked to the interactive ability of the inhibitor. It is useful to predict adsorption centers in the molecule that has a higher possibility for interaction with the metallic surface. The respective geometry of the studied Trizma molecule obtained by optimization was presented in Fig. 1 and it is obvious that it displayed planar configurations.

In previous work, the inhibitive action of Trizma for the acidic corrosion of aluminum in HCl solution has been studied using weight loss method, potentiodynamic and electrochemical impedance spectroscopy (EIS) techniques. The results showed that, Trizma inhibitor has retarding behavior for both the general and pitting corrosion of aluminum (1 × 10^−2^ M Trizma has more than 95% inhibition)^24^.

The choice of substrate due to the importance of carbon steel in most important industries and this medium has chosen to simulate reality where steel undergoes acid cleaning processes by diluted hydrochloric acid. Therefore, this treatment was done to decrease the corrosion rate of steel. Consequently, improving its performance in this medium.

The novality of this work and its aim is to study the inhibitive action of Trizma on the acidic corrosion of mild steel in 1 M HCl solution, using the chemical technique, weight loss method, electrochemical techniques, electrochemical impedance spectroscopy (EIS), and potentiodynamic polarization techniques. Computational calculations have been done to study the performance of Trizma as a corrosion inhibitor. Langmuir, Flory–Huggins isotherm, and the Kinetic–thermodynamic model are applied to test the experimental results and study the type and the mechanism of the Trizma adsorption on the steel surface. Finally, the surface techniques, XRD, SEM, and EDX have been used to study the morphology of the steel surface after corrosion in 1 M HCl solutions without and with different concentrations of Trizma inhibitor, to confirm the other experimental results.

## Experimental

### Materials and solutions

The stock solutions were prepared from analytical grade reagents and distilled water: 37% HCl, and Trizma was purchased from Aldrich chemicals. Trizma is soluble in water by 666 mg/L yielding a clear, colorless solution^25^. It used to prepare 0.05 M stock solution. Before each experiment, a definite volume of 5.0 M HCl is added to an appropriate volume of 0.05 M Trizma solution and diluted with double distilled water to obtain a solution of 1 M HCl and the required concentration of Trizma. The concentration ranges used of Trizma were 1 × 10^–5^ to 1 × 10^−2^ M.

### Weight loss measurements

In these experiments, 2 cm^2^ rectangular mild steel coupons were used with the same chemical composition of samples used in the electrochemical measurements. The chemical compositions of coupons are (wt. %): 0.21 C, 0.35 Si, 0.04 P, 2.5 Mn, 0.04 S, and the remainder iron (96.86). These coupons were polished by abrading with a series of emery papers 400, 600, 800, and 1000 grades, and cleaned with ethanol. After weighing accurately, the specimens were suspended in 100 ml beakers containing 1 M HCl at 30 °C without and with different concentrations of Trizma. After a definite time, the coupons were removed from the solution, washed with distilled water, and ethanol, and then dried with acetone and reweighted. The weight loss was then determined (g/cm^2^) the experiment was then repeated for a different time in travels up to 24 h. To test the reliability and responsibility of the measurements, duplicate experiments were performed in each case under the same conditions. Corrosion rates (weight loss per cm^2^ per hour) were calculated. The results were consistent within 2%.

### Electrochemical tests

Electrochemical impedance and polarization measurements were achieved by using a frequency response analyzer (FRA)/potentiostat supplied from Parstate Instrument (PARSTAT 2263.02 SN 194). The frequency range for EIS measurements was 0.1 × 10^4^ Hz with an applied potential signal amplitude of 10 mV around the resting potential. A three-electrode mode cell contains an auxiliary graphite electrode and a saturated calomel reference electrode was used. The working steel electrode was fabricated in a cylindrical form and encapsulated in epoxy resin in such a way that only one surface of area 0.2728 cm^2^ was left uncovered and thus avoiding the crevice effect. To prevent the migration of any electrolyte, the Teflon gasket thereby forms a watertight seal with the electrode sample. The procedure is similar to the previous work ^26^. The exposed area was mechanically polished with a series of emery papers of variable grades, the samples were then washed thoroughly with distilled water followed by analytical grade ethanol, and finally with distilled water, just before insertion in the cell. Measurements were done at 30 °C. The initial thinking to work in the research is to measure at room temperature, but due to the instability of the room temperature daily at the same degree, 30 °C was fixed to obtain accurate results.

### Scanning Electron Microscope (SEM), Energy Dispersive X-ray Spectrometer (EDS), and X-ray diffraction (XRD) characterizations

One-cm^2^ mild steel coupons were immersed in a test solution without and with various concentrations of Trizma for up to 24 h. By, after 24 h, the coupons were taken out and dried. The nature of the surface film formed on the mild steel coupons surface was examined using the energy dispersive X-ray spectrometer model (EDS, JEM-2100, Japan), X-ray diffractometer, Model (Phillips) X’pert, and JEOL (JSM 6390) Scanning Electron Microscope.

### Density functional theory (DFT) calculations

The theoretical investigations of the Trizma molecule were performed with complete geometry optimization by Gaussian 03 W software, using DFT/B3LYP with a 6-31d basis set in the gas and aqueous phases. The geometry optimization was considered to be complete when the stationary point is located. Among the quantum parameters, the energies of the highest occupied (E_HOMO_) and lowest unoccupied (E_LUMO_) molecular orbitals, energy gap (∆E), dipole moment (μ), electron affinity (A), ionization potential (I), global hardness (η), chemical softness (S) and a fraction of electrons transferred (∆N) were considered and discussed. Quantum parameters were estimated as in the following Equations ^27^:

1 $$ {\text{I}} = - {\text{E}}_{{{\text{LUMO}} }} $$

2 $$ {\text{A}} = - {\text{E}}_{{{\text{HOMO}}}} $$

3 $$ \chi = \left( {{\text{I}} + {\text{A}}} \right)/{2} $$

4 $$ \eta = \left( {{\text{I}}{-}{\text{A}}} \right)/{2} $$

5 $$ {\text{s}} = {1}/\eta $$

The number of transferred electrons (**∆**N) is calculated by application of the Pearson method using the following equation ^28^:

6 $$ \Delta {\text{N}} = (\chi_{{{\text{Fe}}}} - \chi_{{{\text{inh}}}} )/{2}\left( {\eta_{{{\text{Fe }} + }} \eta_{{{\text{inh}}}} } \right) $$

The theoretical values of χFe and ηFe, are 7 eV·mol^−1^ and 0 eV·mol^−1^, considering the metallic surface where iron is the major constituent ^29,30^.

## Results and discussion

### X-ray diffraction results (XRD)

**Figure 2:**
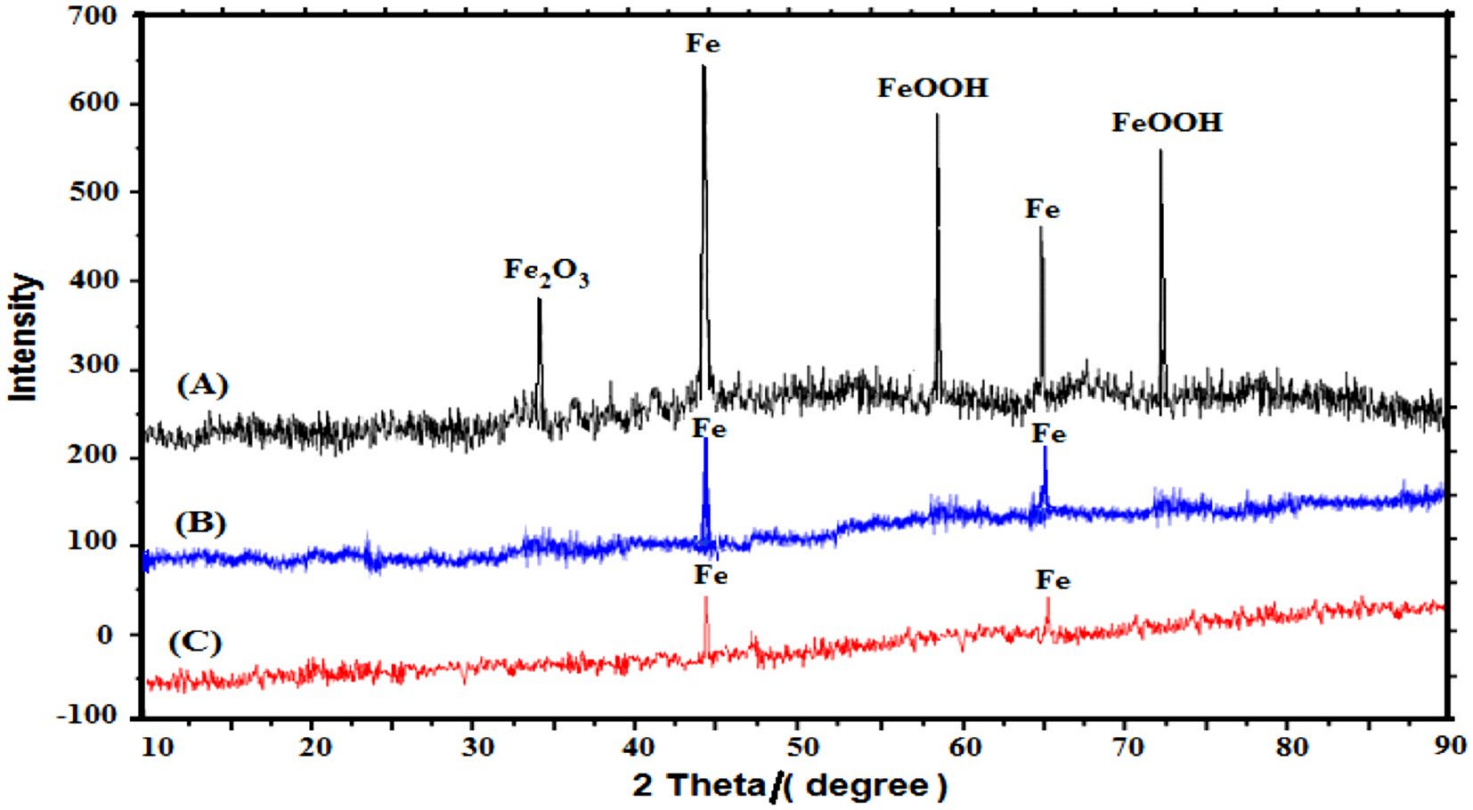
XRD spectrum for mild steel immersed for 48 h in (**A**): 1 M HCl, (**B**): 1 M HCl + 1 × 10^–3^ M Trizma and (**C**): 1 M HCl + 1 × 10^–2^ M Trizma.

The XRD pattern of mild steel surface immersed in 1 M HCl without and with 1 × 10^–3^ M or 1 × 10^–2^ M Trizma for 48 h is shown in Fig. 2. In absence of Trizma, the XRD pattern of the mild steel surface (A) shows the peaks of iron besides another peak at 2*θ* = 25.1°, 49.3°, and 64.6° corresponding to the presence of iron oxides (Fe_2_O_3_ and FeOOH) as corrosion products of steel. However, in presence of Trizma, the XRD patterns of the surface of mild steel (B,C) shows only the peaks of iron and the peaks of the oxides disappear indicating that the presence of Trizma in the medium completely protects the steel from the acidic corrosion.

### SEM study and EDX analysis

**Figure 3:**
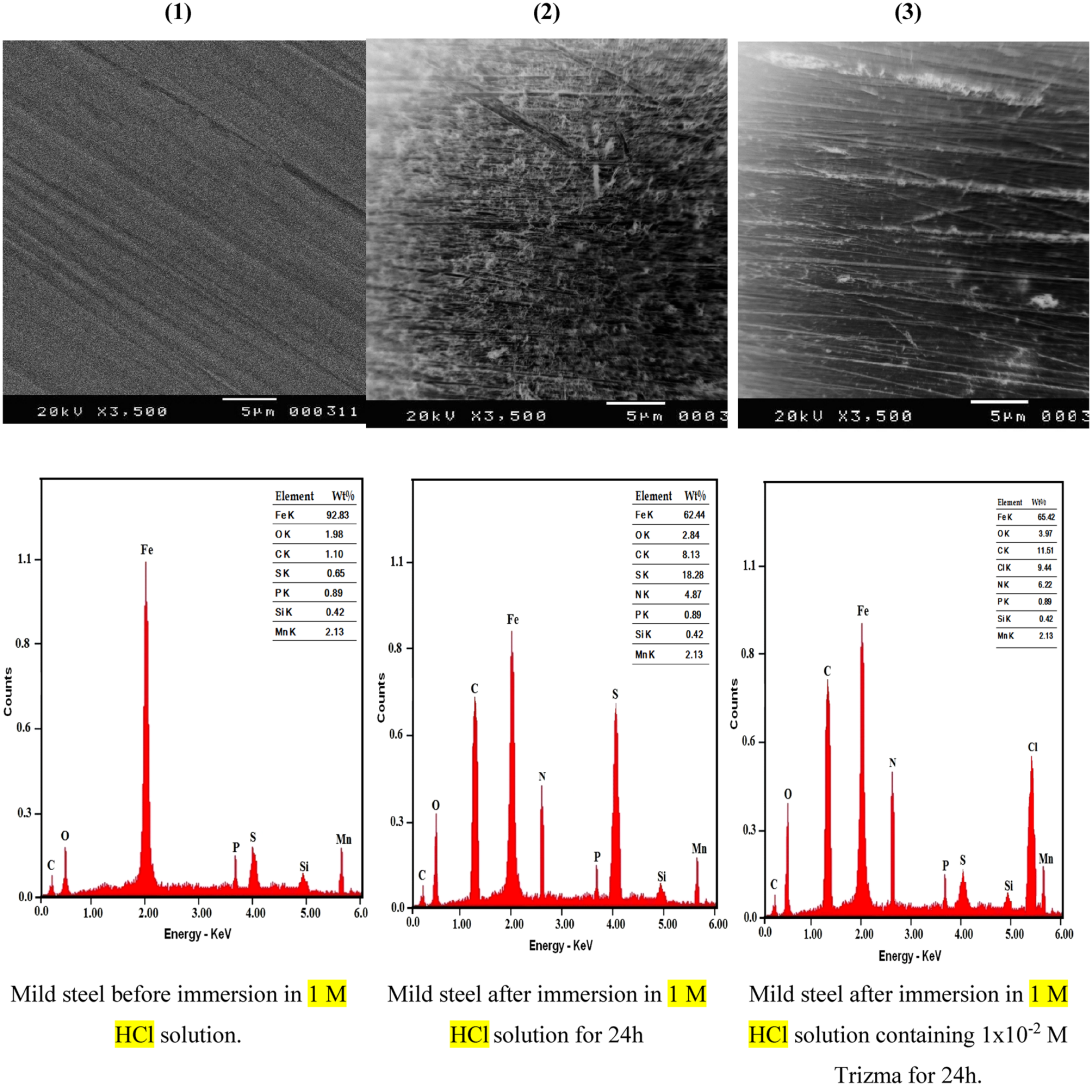
SEM images and EDX micrographs of mild steel before and after immersion in 1 M HCl solution for 24 h in absence and presence of 1 × 10^−2^ M Trizma.

Figure 3 shows the micrographs of each SEM and EDX of mild steel samples before and after immersion in 1 M HCl for 24 h without and with 1 × 10^–2^ M Trizma. Clear polished marks on the surface of the steel before immersion have appeared in the SEM micrograph “1” which shows that the whole surface is smooth and homogeneous. Micrograph “2” shows the mild steel surface after immersion in 1 M HCl for 24 h which was observed as rough and damaged, indicating a vigorous corrosive attack by the acid on the surface. However, Micrograph “3” represents the mild steel surface after immersion in 1 M HCl solution in presence of 1 × 10^–2^ M Trizma—which indicates that Trizma largely inhibited the acidic corrosion of steel and polished marks of the steel surface appears. The EDX results are represented in Table 1, which shows the values of Wt % of nitrogen, oxygen, and carbon on the surface of the steel immersed in 1 M HCl solution without and with 1 × 10^–2^ M Trizma.

**Table 1:** Wt % of N, O and C on the mild steel surface which immersed in 1 M HCl for 24 h in absence and presence of 1 × 10^–2^ M Trizma.

Type	Wt % N	Wt % O	Wt % C
Mild steel	–	1.98	1.10
Mild steel + 1.0 M HCl	–	1.67	1.10
Mild steel + 1.0 M HCl + 1 × 10^–2^ M Trizma	6.22	3.97	11.51

As given in the table, the surface of the steel before and after immersion in free acid solution is free from nitrogen and contains 1.98 Wt% O and 1.10 Wt% C, however, in presence of Trizma, the surface of the steel contains 6.22 Wt% N, 3.97 Wt% O and 1.51 Wt% C. the presence of nitrogen on the surface and increase the amount of each of oxygen and carbon in presence of Trizma in the corrosion environment indicated that this compound is adsorbed on the surface of steel and protected it from corrosion.

### Quantum chemical calculations

#### Global molecular reactivity of Trizma molecule

**Figure 4:**
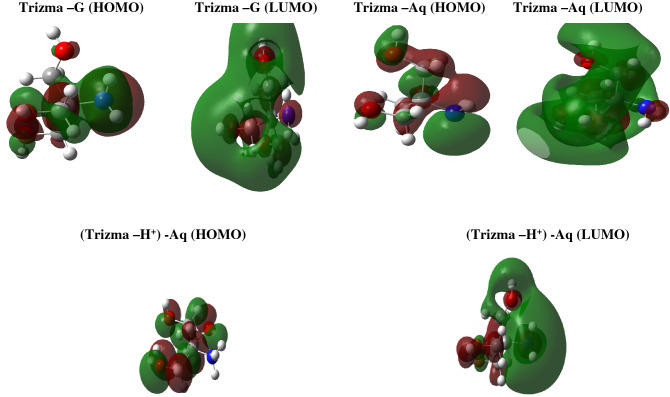
Frontier molecular orbital diagrams (HOMO and LUMO) of studied Trizma molecule.

The relationship between inhibitor efficiency and molecular reactivity has been evaluated by the calculation of quantum chemistry by studying the ability of inhibitor molecules to donate or accept electrons. For this purpose, the studied parameters are E_HOMO_, E_LUMO,_ the energetic gap between MO, and the dipole moment. It is known that the tendency of a molecule to donate electrons is shown by E_HOMO_. With, the high tendency of inhibitor donation of electrons to electron-accepting species with low energy unoccupied molecular orbitals, the higher value of E_HOMO._ Conversely, E_LUMO_ the lowest its value the higher the electron accepting ability of the molecule. Figure 4 represents the Frontier molecular orbital (HOMO and LUMO) of the studied Trizma molecule. In our investigated molecular structure, HOMO density is localized almost in all oxygen atoms in hydroxyl groups. The density of LUMO is almost localized in the whole molecule which is the suitable region for accepting the electrons during the interactions between mild steel and Trizma molecule^31^.

The global indices for chemical reactivity of the Trizma molecule are presented in Table 2. The calculated energies of the frontier orbitals show that the inhibitor molecule has a greater value of energy gap in solution than in the gaseous phase pointing out high electronic stability and less reactivity in solution media. For the Trizma molecule, the high tendency toward heteroatoms protonation is suggested in an acidic solution.

**Table 2:** Computational parameters for neutral as well as protonated form of Trizma obtained with DFT method.

Medium	E_HOMO_ (eV)	E_LUMO_ (eV)	∆E_L-H_ (eV)	μ (Debye)	polarizability α	E_T_ (eV)
**DFT parameters for neutral form of Trizma**						
Gas	− 6.430	0.911	5.519	0.773	64.091	− 439.366
water	− 6.770	0.957	5.813	1.104	79.580	− 439.383
**DFT parameters for Protonated form of Trizma**						
water	− 7.921	− 0.001	7.920	8.269	74.963	− 439.828

The measure of dipole moment (μ) provides several information about the inhibition processes. This parameter reflects the overall molecule polarity. It is the product of the charge on the atoms and the distance between the two bound atoms. Finally, it describes the electronic distribution of molecules. Its high value means that the molecule tends to form the force of Vander Waals from type dipole–dipole interactions with the metal surface, this leads to the form of a good layer of adsorption onto the metal surface then gives high inhibition efficiency^32–35^. Therefore, the inhibition ability toward corrosion can be explained by increasing the volume of the inhibitor molecule which increases the adsorption area between the inhibitor and the metal surface. As obtained in Table 2, μ increased significantly in water than in the gaseous phase for a neutral form of Trizma molecule. This high value means increasing the adsorption strength between the inhibitor and the metal surface. Also, a comparison between both neutral and protonated species reveals that μ is higher for the protonated molecule than for the non-protonated molecule indicating that the neutral molecule is less likely to adsorb to the surface of mild steel than the protonated species.

Besides the strong dipole–dipole interaction between inhibitor and surface of the metal, the high value of μ also suggests that it is a polar compound and can easily donate electrons to form dπ-pπ bonding^36^.

Dipole polarizability α is also represented in Table 2. Higher values of it show a strong adsorption process.

Electronegativity (*X)* is considered the power of an electron or group of atoms to attract electrons toward itself^37^. According to Sanderson’s principle of electronegativity equalization, the electronegativity becomes adjusted to the intermediate value when two or more atoms come together to form a molecule. Since the ability to donate electrons to the metallic surface regards the good inhibitors, it is expected that increasing the inhibitive efficiencies is connected with the decrease of electronegativity values. The values of *X* of the studied molecule are calculated and presented in Table 3.The obtained values indicate the strong donation from the inhibitor to the metal surface.

**Table 3:** Calculated quantum chemical descriptors for neutral as well as protonated form of Trizma obtained with DFT method.

Medium	I (eV)	A (eV)	*X* (eV)	η (eV)	σ (eV)	∆N
**DFT parameters for neutral form of Trizma**						
G	6.430	− 0.911	2.760	3.670	0.272	0.578
A	6.770	− 0.957	2.907	3.864	0.259	0.530
**DFT parameters for Protonated form of Trizma**						
A	7.921	0.001	3.961	3.960	0.253	0.384

Lukovits et al. study^38^ mention that the precise term referring to ∆N “electron-donating ability”, does not imply the representation of the number of electrons leaving the donor and entering the acceptor molecule. But, indicates the tendency of a molecule to donate electrons to the metal surface. The obtained values of ∆N reported in Table 3, show that the value of ∆N (0.578 eV) is less than 3.6 eV, indicating that the studied Trizma tends to donate electrons to the steel surface.

From (Tables 2 and 3) in gas and aqueous phases, the computed results showed that the decrease of the total energy (E_T_) compared to the calculated energy in the gas phase indicates the stabilization effect of the solvent. Also, noticed an increase in the charge separation in the molecules that were inferred from high values of the dipole moment μ in solution.

#### Molecular electrostatic potential effect (MEP)

**Figure 5:**
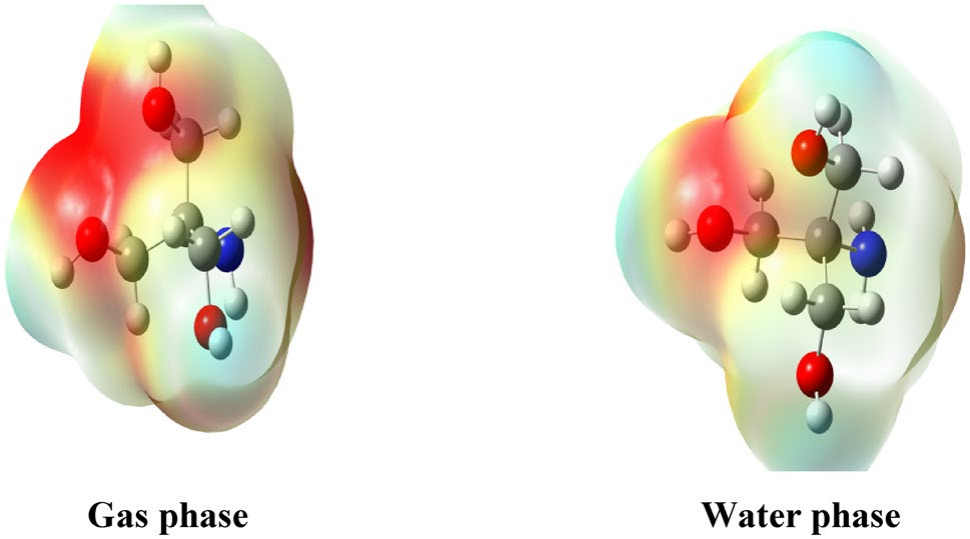
Molecular electrostatic potential (MEP) of the Trizma molecule.

The molecular electrostatic potential representation of the Trizma molecule was determined to identify regions of electron density. MEP maps for the Trizma molecule are shown in Fig. 5.

According to the literature, blue and red signified areas of strong positive and negative electrostatic potential respectively, such that the electrostatic potential decreased in the following order: blue, green, orange, and red ^39,40^. In this study, the highest electron density (red color) resides on the two oxygen atoms (O14, O16) and the lowest electron density is located on the nitrogen atom (N) and some carbon atoms.

#### Local molecular reactivity of Trizma molecule

**Figure 6:**
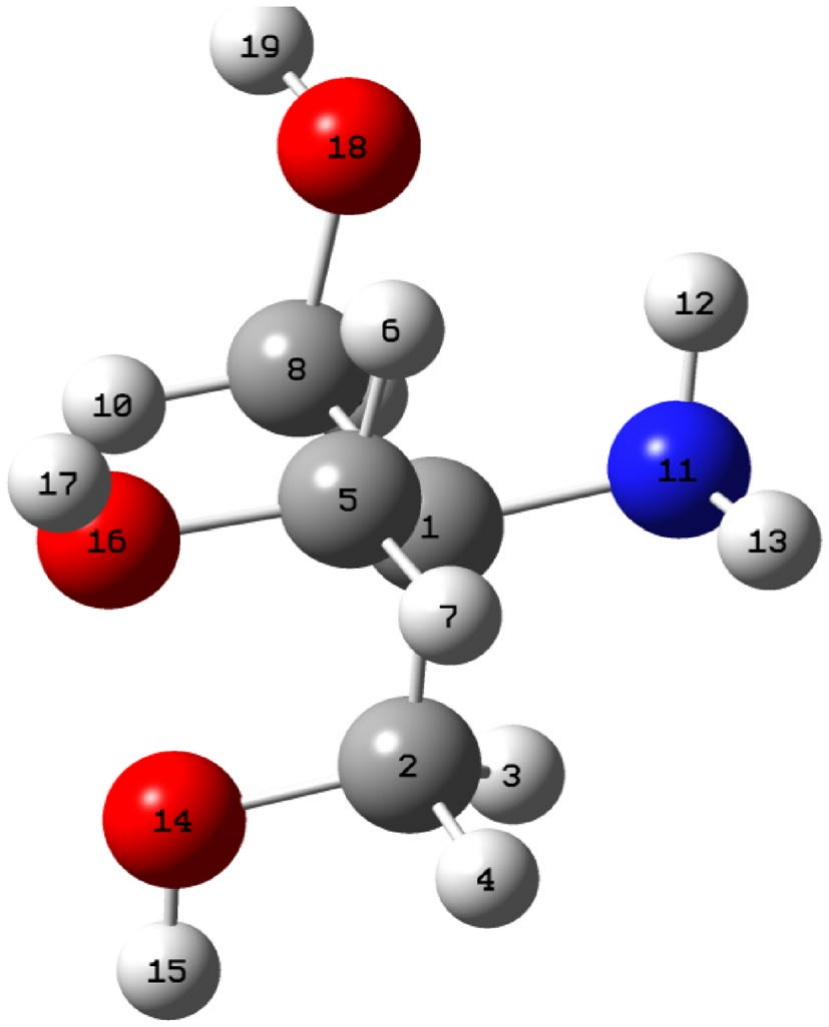
Fukui indices of the studied Trizma molecule.

Figure 6 shows the sites of reactivity for a neutral form of the studied Trizma molecule which has been identified by calculating Fukui functions. There were calculated using a finite difference approximation as the following equations ^41^:

$$ {\text{Nucleophilic attack}}:\quad {\text{f}}^{ + }_{{\text{k}}} = {\text{q}}_{{\text{k}}} \left( {{\text{N}} + {1}} \right) - {\text{q}}_{{\text{k}}} \left( {\text{N}} \right) $$

$$ {\text{Electrophilic attack}}: \quad {\text{f}}^{ - }_{{\text{k}}} = {\text{q}}_{{\text{k}}} \left( {\text{N}} \right) - {\text{q}}_{{\text{k}}} \left( {{\text{N}} - {1}} \right) $$

where qk(N), qk(N + 1), qk(N − 1) are the charge values of an atom k for the neutral, the anion, and the cation, respectively.

It is known that the most susceptible site to nucleophilic attack is the place where f_k_^+^ displays its maximum value, whereas the best site for electrophilic attack is defined by the maximum value of f_k_^–^^42,43^.

From the data in Table 4, it can be noticed that the C2,C5, C8, O14 and O16 atoms are favorable sites for nucleophilic attack due to their high values of f ^+^ for the studied Trizma molecule, which enhances the adsorption of this compound on the metal surface. These nucleophilic centers appear red and electrophilic centers are in pale blue as mentioned in Fig. 6.

**Table 4:** The most relevant values of the natural population with the corresponding values of the Fukui functions of the neutral studied form of Trizma inhibitor in gas phase.

Atom	qk(N + 1)	qk(N)	qk(N − 1)	f ^+^	f ^− ^	∆f
C_1_	0.001	− 0.016	− 0.103	0.017	0.057	− 0.04
C_2_	− 0.076	− 0.140	− 0.146	0.064	− 0.006	0.07
C_5_	− 0.124	− 0.156	− 0.171	0.032	0.015	0.02
C_8_	− 0.121	− 0.160	− 0.162	0.039	0.002	0.04
N_11_	− 0.735	− 0.756	− 0.542	0.021	− 0.214	0.24
O_14_	− 0.523	− 0.557	− 0.470	0.034	− 0.087	0.12
O_16_	− 0.532	− 0.555	− 0.447	0.023	− 0.108	0.13
O_18_	− 0.562	− 0.594	− 0.572	0.032	− 0.022	0.05

### Electrochemical impedance spectroscopy (EIS) results

**Figure 7:**
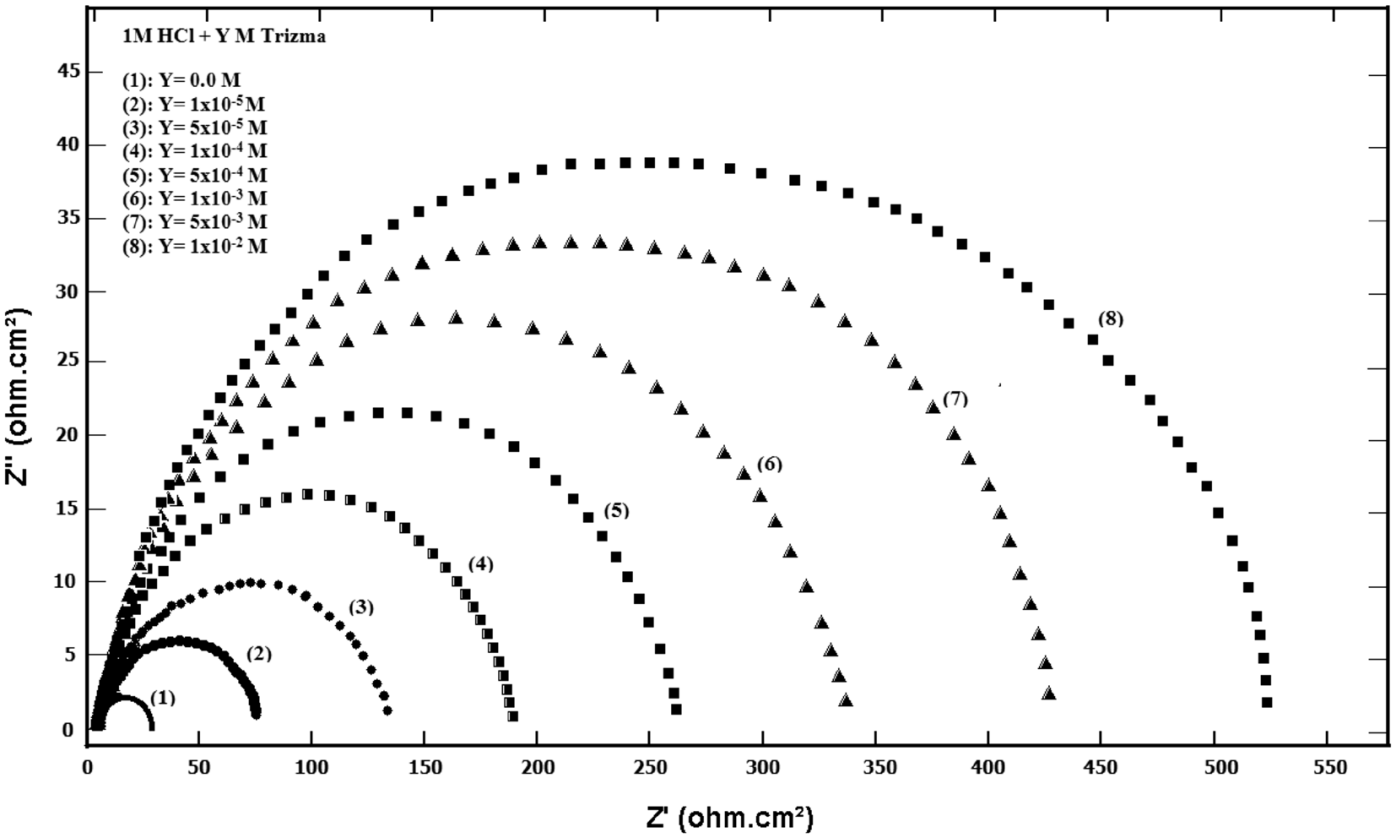
Nyquist plots for mild steel in 1 M HCl solution without and with various concentrations of Trizma at 30 °C.

**Figure 8:**
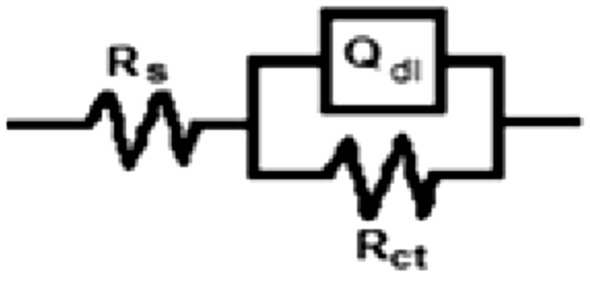
Schematic for the equivalent circuit of mild steel.

The Nyquist plots of steel in 1 M HCl solution without and with various concentrations of Trizma are given in Fig. 7 that shows a depressed capacitive semicircle indicating that the dissolution process of steel takes place under activation control. The impedance spectra were analyzed by using a simple equivalent circuit model (Fig. 8) which includes the solution resistance *R*_*s*_ and the double layer capacitance (*Q*_*dl*_) placed parallel to the charge transfer resistance element (*R*_*ct*_) which indicates the electron transfer across the surface, and its value proportional to the corrosion rate inversely.

Table 5 shows the values of *R*_*s*_*, R*_*ct*_*, C*_*dl*_*,* and the *% P* obtained from EIS results for mild steel in 1 M HCl solution without and with various concentrations of Trizma. The values of *% P* are calculated using the equation:

7 $$ \% P = \left[ {\left( {R_{ct} - R_{cto} } \right)/R_{ct} } \right] \times {1}00 $$

where *R*_*cto*_ and R_ct_ are the charge transfer resistances, without and with Trizma inhibitor respectively.

**Table 5:** Corrosion parameters obtained from impedance curves of mild steel in 1 M HCl solution containing various concentrations of Trizma at 30 °C.

Conc., mole/L	*R*_*s*_ (Ohm.cm^2^)	*Q*_*dl*_ (µF/cm^2^)	*n*	*R*_*ct*_ (Ohm.cm^2^)	*% P*
0.0	0.11 ± 0.001	193	0.9 ± 0.001	33.4 ± 0.08	–
1.0 × 10^–5^	0.24 ± 0.002	176	0.9 ± 0.002	74.3 ± 0.09	55.0 ± 0.13
5.0 × 10^–5^	0.19 ± 0.001	142	0.9 ± 0.001	142.5 ± 0.10	76.6 ± 0.25
1.0 × 10^–4^	0.23 ± 0.001	136	0.9 ± 0.003	192.7 ± 0.11	82.7 ± 0.38
5.0 × 10^–4^	0.26 ± 0.002	129	0.9 ± 0.001	260.6 ± 0.13	87.2 ± 0.63
1.0 × 10^–3^	0.28 ± 0.001	121	0.9 ± 0.002	347.3 ± 0.15	90.4 ± 0.88
5.0 × 10^–3^	0.29 ± 0.001	113	0.9 ± 0.003	442.7 ± 0.18	92.5 + 1.26
1.0 × 10^–2^	0.26 ± 0.002	104	0.9 ± 0.001	526.2 ± 0.19	93.7 ± 1.38

± shows the standard deviation of all measurements.

**Figure 9:**
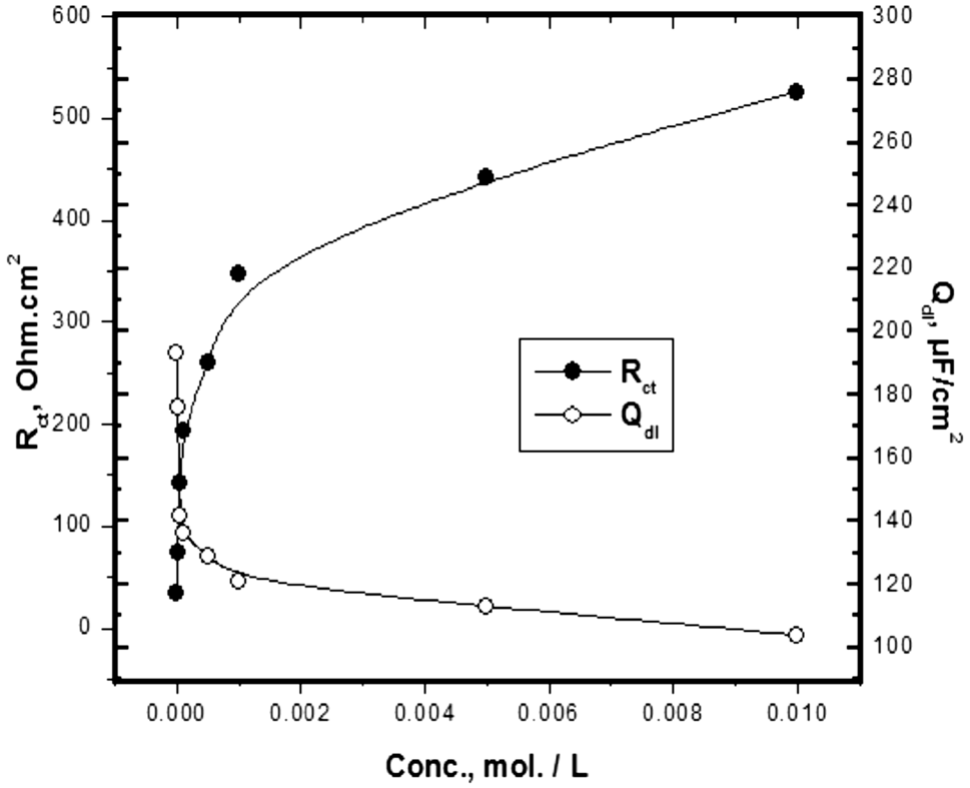
Variations of the *R*_*ct*_ and *C*_*dl*_ values with the concentrations of Trizma at 30 °C.

The effect of Trizma concentration on each *R*_*ct*_ and *C*_*dl*_ of mild steel in 1 M HCl solution is given in Fig. 9. The data show an increasing the *R*_*ct*_ values and a decrease in the value of *C*_*dl*_ with increasing the concentration of Trizma, *n* equal 0.9 with all concentrations of Trizma. This behavior indicates that the presence of Trizma in the solution retards the steel corrosion due to the adsorption of its molecules on the surface of the steel.

The presence of 1 × 10^–2^ M Trizma in the acid solution gives 93.7% inhibition indicating that Trizma act as an efficient eco-friendly inhibitor for the acidic corrosion of steel.

### Potentiodynamic polarization results

**Figure 10:**
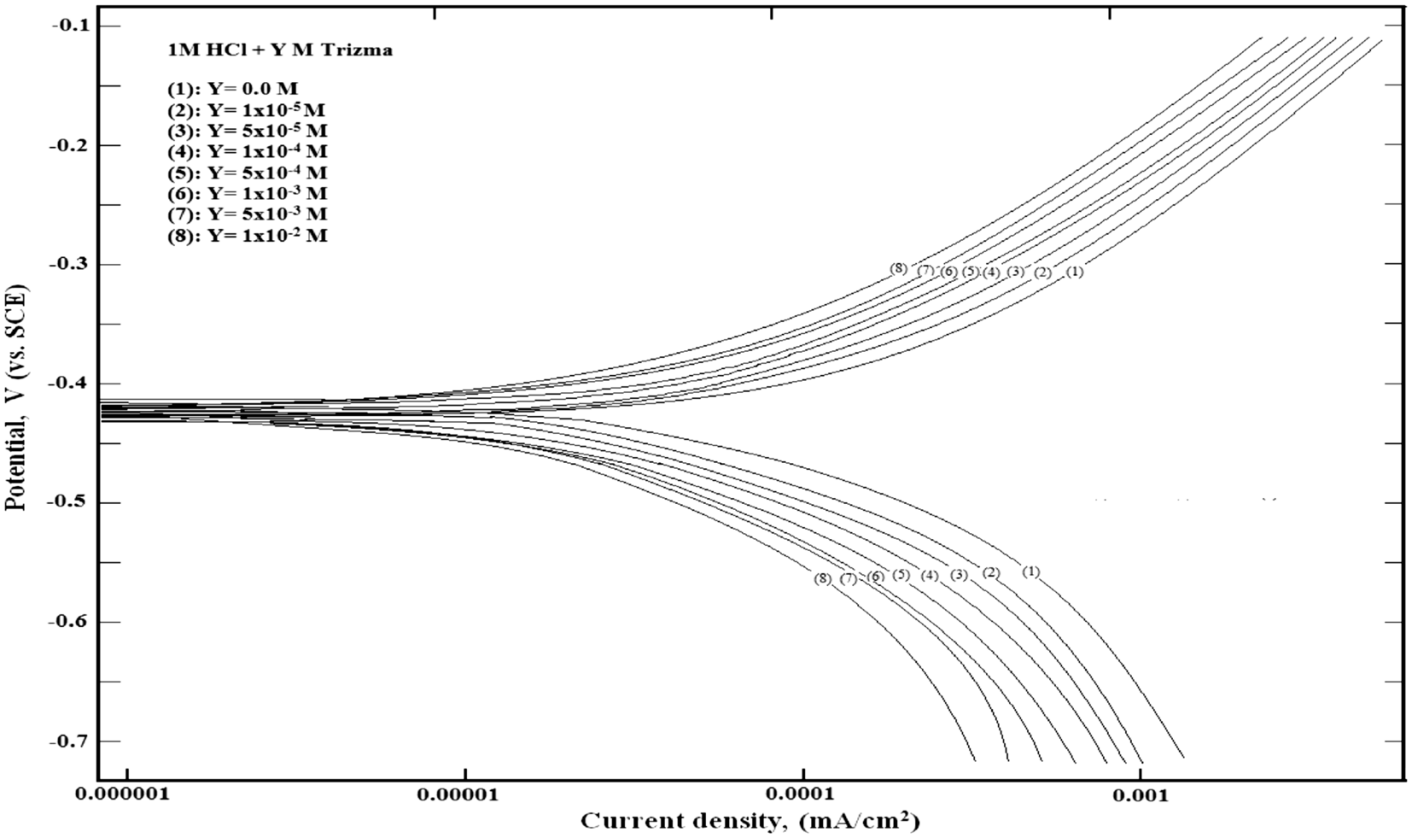
Polarization curves for mild steel in 1 M HCl solution without and with various concentrations of Trizma at 30 °C.

Figure 10 shows the polarization curves of mild steel in 1 M HCl solution without and with different concentrations of Trizma. Trizma behaves as a mixed-type inhibitor for the acidic corrosion of steel.

The values of the electrochemical parameters obtained from polarization curves of steel in 1 M HCl solution without and with various concentrations of Trizma are given in Table 6. The data show the observed decrease in the corrosion current density (*i*_*corr*_) and a slight shift in the corrosion potential (*E*_*corr*_) to more negative potentials by increasing Trizma concentration pointing that it could be used as a pickling inhibitor^44^. Cathodic Tafel slope (*β*_*c*_) decreased with increasing the concentration of Trizma. On the other hand, the anodic Tafel slope (*β*_*a*_) increased with increasing the concentration of Trizma.

**Table 6:** Corrosion parameters obtained from potentiodynamic polarization curves of mild steel in 1 M HCl containing different concentrations of Trizma. ± shows the standard deviation of all measurements.

Conc., mole/L	− *E*_*corr.*_ (mV vs. SCE)	*β*_*a*_	− *β*_*c*_	*i*_corr_*.* (mA cm^−2^)	%*P*
		(mV.decade^−1^)			
0.0	**418** ± 1.10	**97** ± 0.48	**128** ± 0.82	**0.1491** ± 1.68	–
1.0 × 10^–5^	419 ± 1.12	94 ± 0.39	126 ± 0.80	0.0699 ± 1.30	53.1 ± 0.23
5.0 × 10^–5^	421 ± 1.11	87 ± 0.36	123 ± 0.77	0.0388 ± 1.28	73.9 ± 0.24
1.0 × 10^–4^	423 ± 1.13	80 ± 0.34	121 ± 0.76	0.0301 ± 1.26	79.8 ± 0.25
5.0 × 10^–4^	425 ± 0.94	76 ± 0.28	119 ± 0.73	0.0229 ± 1.24	84.6 ± 0.26
1.0 × 10^–3^	426 ± 1.12	68 ± 0.25	117 ± 0.71	0.0179 ± 1.28	87.9 ± 0.24
5.0 × 10^–3^	428 ± 1.14	57 ± 0.27	114 ± 0.68	0.0159 ± 1.32	89.3 ± 0.21
1.0 × 10^–2^	433 ± 1.11	49 ± 0.23	111 ± 0.64	0.0129 ± 1.38	91.3 ± 0.18

± shows the standard deviation of all measurements.

The percentage inhibition of Trizma (*%P*) was calculated from the polarization measurements using the following equation:

8 $$ \% P = \left[ {\left( {i_{o} {-}i} \right)/{\text{i}}_{{\text{o}}} } \right] \times {1}00 $$

where i_o_ and i are the corrosion current density without and with different concentrations of Trizma. The results clarify that *% P* increases with increasing the Trizma concentration and reaches 91.3% in presence of 1.0 × 10^–2^ M is in good agreement with the value obtained from EIS results and confirms that Trizma behaves as an efficient Eco-friendly inhibitor for the acidic corrosion of steel.

### Weight loss results

**Figure 11:**
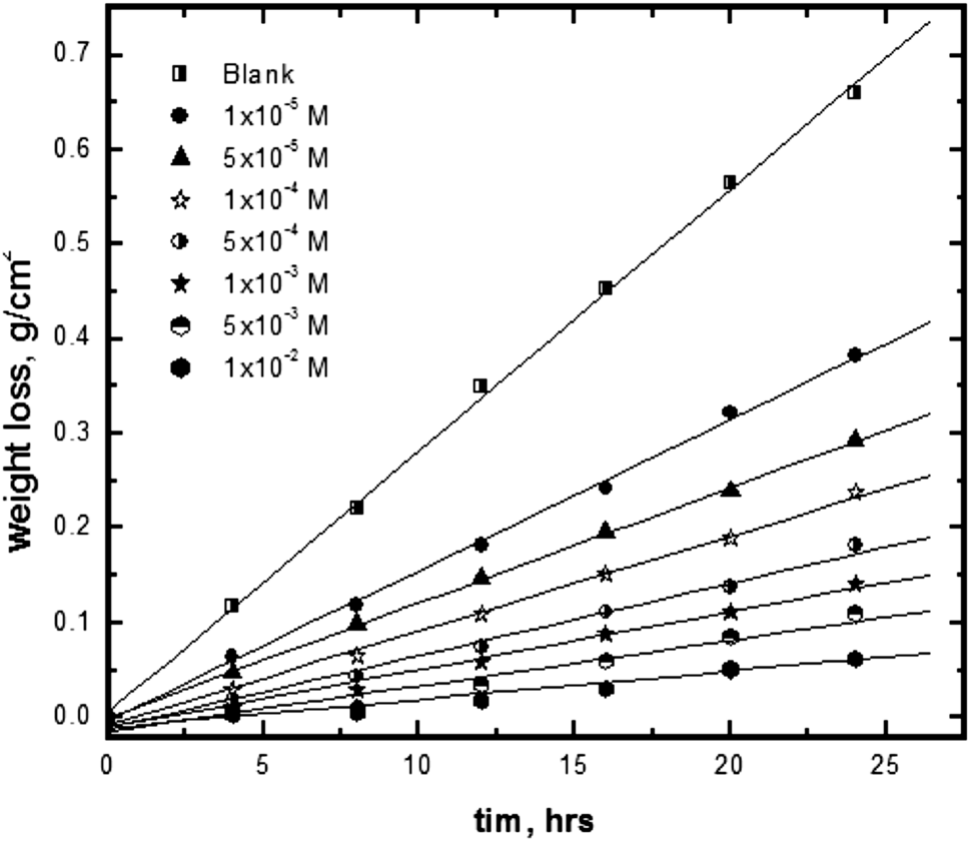
Variation of mild steel weight loss against immersion time in 1 M HCl without and with different concentrations of Trizma at 30 °C.

The variation of weight loss of mild steel in 1 M HCl, without and with various concentrations of Trizma with exposure time up to 24 h at 30 °C are shown in Fig. 11. As shown, the weight loss increased with exposure time and the rate of corrosion is the slope of the straight lines. The slope of the straight lines decreased sharply with the addition of the Trizma. The percentage inhibition (*% P*) was calculated by the following Equation:

9 $$ \% P = \left[ {\left( {{\text{R}}_{{\text{o}}} - {\text{R}}} \right)/{\text{R}}_{{\text{o}}} } \right] \times {1}00 $$

where, R_o_ and R are the values of corrosion rate (g.cm^-2^.hr^-1^) without and with Trizma inhibitor, the results of corrosion rates of mild steel and the corresponding percent inhibition of Trizma are given in Table 7. The data show that the percent inhibition of Trizma increases by increasing its concentration and reaches 88.1% in presence of 1.0 × 10^–2^ M. this value is in fair agreement with those obtained from the potentiodynamic polarization and EIS results confirming that Trizma behaves as a good eco-friendly inhibitor of the acidic corrosion of steel. This study, within 24 h as immersion time, showed an inhibition efficiency up to 88%, this time is sufficient to ensure the effectiveness of the inhibitor.

**Table 7:** Corrosion parameters obtained from weight loss of mild steel in 1 M HCl solutions containing various concentrations of Trizma at 30 °C.

Conc., mol. /L	*R* (g cm^−2^ hr^−1^ )	% *P*
0.0	0.02773 ± 0.14	–
1.0 × 10^–5^	0.01596 ± 0.12	42.4 ± 0.14
5.0 × 10^–5^	0.01216 ± 0.11	56.1 ± 0.21
1.0 × 10^–4^	0.01000 ± 0.10	63.9 ± 0.29
5.0 × 10^–4^	0.00763 ± 0.13	72.5 ± 0.07
1.0 × 10^–3^	0.00612 ± 0.11	77.9 ± 0.21
5.0 × 10^–3^	0.00480 ± 0.10	82.7 ± 0.29
1.0 × 10^–2^	0.00353 ± 0.08	88.1 ± 0.43

± shows the standard deviation of all measurements.

### Application of adsorption isotherms

There are many adsorption isotherms are uses in literatures, but we use only three adsorption isotherms:-

1. Langmuir isotherm to identify the ideal adsorption of inhibitor molecules on the metal surface.
2. Flory–Huggins isotherm to measure the number of adsorbed water molecules displaced by a studied inhibitor molecule from the metal surface.
3. Kinetic—thermodynamic model to calculate the number of active sites of the metal surface occupied by single molecule of the inhibitor.

Langmuir, Flory Huggins isotherms, and Kinetic-thermodynamic models were used to fit the corrosion inhibition data of Trizma.

The Langmuir isotherm is represented by^45^

10 $$ [\theta /({1} - \theta )\left] { = {\text{K}}} \right[{\text{C}}] $$

where K is the binding constant representing the interaction of the inhibitor with the metal surface and C is the inhibitor concentration.

Flory–Huggins isotherm given with^46^

11 $$ \theta /\left[ {{\text{x}}\left( {{1} - \theta } \right)^{{\text{x}}} } \right] = {\text{K}}\left[ {\text{C}} \right] $$

where x is the size parameter and is a measure of the adsorbed water molecules number displaced by a studied inhibitor molecule from the metal surface.

The Kinetic—thermodynamic model given by^47^

12 $$ {\text{Log }}[\theta /({1} - \theta )] = {\text{Log K}}^{\prime } + {\text{yLog C}} $$

where y is the number of the molecules of the inhibitor occupying one active site of the metal surface and (1/y) represents the number of active sites occupied by one inhibitor molecule. The binding constant K is given by:

13 $$ {\text{K}} = {\text{K}}^{{\prime}{({1}/{\text{y}})}} $$

**Figure 12:**
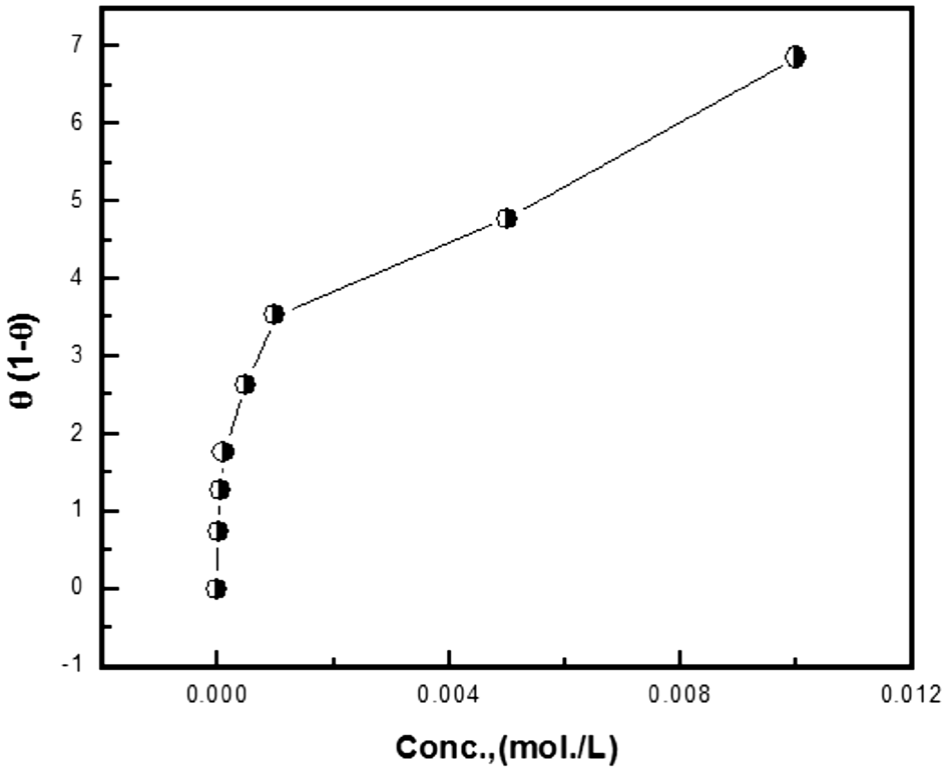
Linear fitting of the data of adsorption of Trizma to Langmuir isotherm for mild steel in 1 M HCl solution.

**Figure 13:**
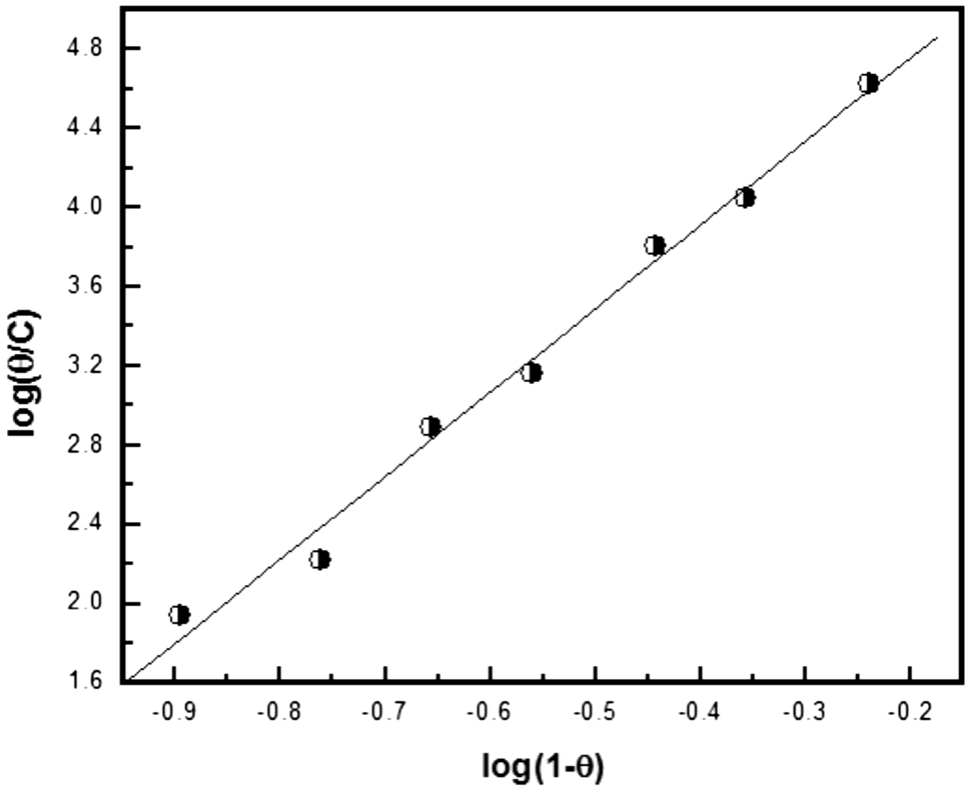
Linear fitting of the data of adsorption of Trizma to Flory–Huggins isotherm for mild steel in 1 M HCl solution.

**Figure 14:**
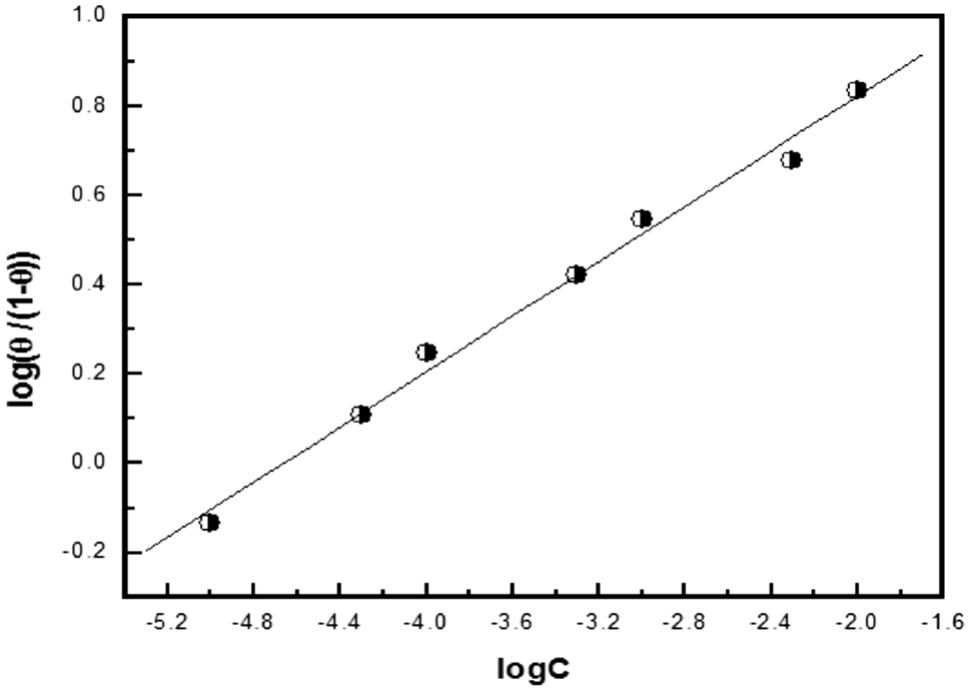
Linear fitting of the data of adsorption of Trizma to Kinetic—thermodynamic model for mild steel in 1 M HCl solution.

Figures 12, 13, 14 show the application of the above-mentioned models to the results of Trizma adsorption on mild steel surface in 1 M HCl solution which was obtained from the weight loss method. The obtained parameters from the figures are depicted in Table 8.

**Table 8:** Linear parameters fitted by the application of the used adsorption models for the corrosion inhibition of mild steel in 1 M HCl with Trizma inhibitor.

Langmuir	Flory- Huggins		*R*^2^	Kinetic-Thermodynamic		*R*^2^
K	x	K		1/y	K	
–	4.2	9.5 × 10^4^	0.9948	3.2	4.6 × 10^4^	0.99512

The Langmuir isotherm does not fit with the experimental results of Trizma indicating that there is non-ideal behavior in the adsorption process of it on the steel surface in 1 M HCl solutions. On the other side, the Flory–Huggins isotherm and Kinetic-thermodynamic model are found to fit the data obtained. The binding constant K values of Trizma with mild steel surface obtained from the two models are in good agreement and give ∆G°_ads_ ~ − 39 kJ/mol, indicating the adsorption process of Trizma on the steel surface is cooperative (chemical–physical). Flory–Huggins isotherm gives *x* = 4 which means that each Trizma molecule displaces four adsorbed water molecules from the steel surface, however, the Kinetic-thermodynamic model gives 1/y = 3 indicating that each Trizma molecule occupies three active sites of the steel surface. In a previous work on the corrosion inhibition of aluminum in 1 M HCl solution by Trizma^24^, the application of the Kinetic-thermodynamic model on the experimental results indicated that ∆G°_ads_ ≈ − 34.9 kJ/mol & and each Trizma molecule occupies two active sites of the aluminum surface. A comparison of Trizma with other inhibitors in previous works has been reported in Table 9.

**Table 9:** Comparison of Trizma inhibitor results with other inhibitors for steel in 1 M HCl.

Inhibitor	Optimum conc	Maximum efficiency, %	References
1-(2-(4-nitrophenyl)-2-oxoethyl)pyridazinium bromide	10^−3^ M	85	^48^
2-(2, 6-dichloranilino) phenyl acetic acid Drugs	2.5%	87.5	^49^
Synthesized Benzimidazole Derivative	10^−4^ M	93	^50^
Alanine	50 mM	80	^51^
Chloroquinolines	5 × 10^−4^ M	94	^52^
Ranitidine	2 × 10^–3^ M	95.53	^53^
Donaxine	7.5 mM	98	^54^
Aminophylline	0.6 mM	87.3	^55^
Trizma	1 × 10^–2^ M	93.7	This work

### Mechanism of inhibition

In presence of Trizma in the corroded medium, SEM images showed better steel surface with less corrosion products and the EDX results confirmed the presence of nitrogen and increase the amount of each of oxygen and carbon on the steel surface indicating that this compound is adsorbed on the steel surface. The XRD pattern of mild steel surface immersed in 1MHCl solution in presence of Trizma shows only the peaks of iron and the peaks of the oxides disappear due to the complete inhibition of the acidic corrosion of steel. The potentiodynamic polarization results showed the Trizma act as a mixed type inhibitor retard both the cathodic and anodic reactions of corrosion due to adsorption of its molecules at both cathodic and anodic area of the steel/solution interface. The electrochemical impedance spectroscopy results showed an increasing the Rct values and a decrease in the value of Cdl with increasing concentration of Trizma indicating that the presence of Trizma in the solution retards the steel corrosion due to the adsorption of its molecules on the surface of the steel. The quantum calculation results confirmed the ability of Trizma molecule to inhibit the acid corrosion of steel. Application of the adsorption isotherms to fit the experimental results of inhibition of Trizma to corrosion of steel in 1MHCl solution showed that Langmuir isotherm is not applicable due to the non-ideal adsorption of Trizma on the steel surface. However each of Flory–Huggins isotherm and the Kinetic—thermodynamic model are applicable and gave ∆G°ads ≈ − 39.0 kJ/mol indicating that the adsorption process is cooperative (chemical-physical) and mainly chemical.

## Conclusion

The results of this study show that:

1. Weight loss, electrochemical, XRD, SCE, and EDX results indicated that Trizma efficiently inhibits the acidic corrosion of steel.
2. The potentiodynamic results indicated that Trizma act as mixed- a type inhibitor.
3. The electrochemical impedance spectroscopy results indicated that the presence of Trizma in solution retards the steel dissolution due to the adsorption of its molecules at the steel solution interface.
4. There is a good agreement between the results of the percent inhibition of Trizma measured using the weight loss, potentiodynamic polarization, and electrochemical impedance techniques.
5. XRD and SEM results pointed out that the presence of Trizma in the corrosion medium completely inhibited the corrosion of steel in the acid solution, mainly due to the non-ideal adsorption of the Trizma molecules at the steel surface.
6. The results of application of the adsorption isotherms to fit the experimental results of the inhibition of the corrosion of steel in 1MHCl solution showed that Langmuir isotherm is not applied, however, Flory–Huggins isotherm and Kinetic- thermodynamic model are applied giving ∆G°_ads_ ~ − 39.0 kJ/mol and the adsorption process of Trizma on the steel surface is cooperative (chemical–physical) mainly chemical adsorption.
7. The quantum calculation results confirmed the high ability of Trizma molecule to inhibit the acidic corrosion of steel.

## Data Availability

All the data presented in the current study are available from the corresponding author on reasonable request.
